# Tripeptidyl Peptidase 1 Regulates Human Trophoblast Cell Proliferation Implying a Role in Placentation

**DOI:** 10.1155/2022/6856768

**Published:** 2022-09-12

**Authors:** Wei Zhou, Evdokia Dimitriadis

**Affiliations:** ^1^Department of Obstetrics and Gynaecology, University of Melbourne, Parkville, Victoria 3010, Australia; ^2^Gynaecology Research Centre, Royal Women's Hospital, Parkville, Victoria 3052, Australia

## Abstract

Proper placentation in the first trimester is essential for a healthy pregnancy in humans. A recent proteomics study of human placental tissue has identified that tripeptidyl peptidase 1 (TPP1) production is reduced in the placenta in early-onset preeclampsia compared to uncomplicated pregnancy. However, it remains to be investigated if TPP1 plays a role in regulating trophoblast cell function during early pregnancy. In this study, immunohistochemistry was used to determine the production and localization of TPP1 in human placenta throughout gestation and the first-trimester decidua/implantation sites. *TPP1* siRNA (20 nM) was transfected into a human trophoblast cell line (HTR8/SVneo) to knock down *TPP1*, and functional consequences on cell adhesion, proliferation, migration, and invasion were analyzed via xCELLigence real-time monitoring. The expression of TPP1 downstream targets was examined by qPCR. Our data show that TPP1 localized to the discrete foci in the cytoplasm in syncytiotrophoblast, cytotrophoblast, and decidual cells across all trimesters of pregnancy. In the first-trimester human decidua, TPP1 exhibited similar staining patterns in the cytotrophoblast cells based at the cell columns. However, minimal/no staining was identified in the HLA-G positive extravillous trophoblast cells (EVTs), especially in the EVTs that invaded in the decidua. Knockdown of *TPP1* in HTR8/SVneo cells by 95% significantly impaired cell adhesion and proliferation without affecting cell migration and invasion. qPCR revealed that the expression of cell proliferation markers *P21* and *MKI67* and *TPP1*-related genes *MRE11*, *CLN3*, and *CLN8* was significantly changed after *TPP1* knockdown in HTR8/SVneo cells compared to control. Overall, our data demonstrate that TPP1 alters trophoblast cell line function suggesting that it may be involved in regulating human placentation in the first trimester via controlling trophoblast cell adhesion and proliferation.

## 1. Introduction

Normal placentation in the first trimester is crucial for a healthy pregnancy. Human placentation initiates after embryo implantation to form a functional placenta. Cytotrophoblast cells in the placental villi form the anchoring chorionic villi, which anchor the placenta to the decidua where they proliferate to form cell columns. Within the columns, the trophoblast differentiates from a non-invasive, proliferative phenotype towards an invasive but less proliferative phenotype [1, 2]. These invasive cells (called extravillous trophoblast [EVT]) migrate and invade into maternal decidua, whereupon they engraft and remodel maternal spiral arteries to create high-flow, low-resistance vessels by the end of the first trimester ensuring blood supply to the placenta [2, 3]. Dysregulation of trophoblast cell adhesion, proliferation, migration, and invasion can lead to dysfunctional placentation and obstetric complications such as preeclampsia [4].

Tripeptidyl peptidase 1 (TPP1) is a lysosomal serine protease widely expressed in higher organisms [5]. Defective TPP1 function results in abnormal accumulation of protein and lipofuscin-like material in cells eventually leading to cell dysfunction [6, 7] and diseases including the neurodegenerative disease neuronal ceroid lipofuscinosis type 2 (CLN2). In a zebrafish model of human CLN2 disease, TPP1 deficiency leads to sustained reduction of cell proliferation in the central nervous system [8]. In hepatocellular carcinoma, knockdown of TPP1 inhibits cell proliferation, migration, and invasion in vitro [9]. Our recent work has demonstrated that TPP1 is expressed at significantly lower levels in human luminal epithelial cells of infertile endometrium compared to fertile. Functional analysis reveals that knockdown of *TPP1* in human endometrial epithelial cells impairs cell adhesive capacity [10]. Since placentation requires highly regulated cell adhesion, proliferation, migration, and invasion, the involvement of TPP1 in mediating these activities support its potential roles in regulating normal placentation.

Although the localization and function of TPP1 in human placenta have not been determined, a recent proteomics analysis of human placental tissue has identified reduced TPP1 expression in the placenta in early-onset preeclampsia compared to uncomplicated pregnancy [11]. This study aimed to determine the localization of TPP1 in human placenta and its function on trophoblast cell (HTR8/SVneo) adhesion, proliferation, migration, and invasion.

## 2. Materials and Methods

### 2.1. Human Placental Tissue Collection

Placental tissues were collected from healthy women undergoing pregnancy termination for psychosocial reasons (first and second trimesters, 7–22 weeks; *n* = 9) and delivery at term (37–42 weeks; *n* = 4) at Monash Health and The Royal Women's Hospital (ethics number: #09317B). Written informed consent was obtained from each patient before surgery. Collected placental tissues were quickly washed with Hanks' Balanced Salt Solution and subjected to 10% formalin fixation for immunohistochemistry.

### 2.2. Antibodies and Cell Line

Mouse monoclonal antibody against TPP1 (#AB54685) was purchased from Abcam (Cambridge, UK), and mouse monoclonal antibody against HLA-G (#557577) was purchased from BD Pharmingen (San Diego, CA, USA). Mouse IgG isotype control (#X0931) was purchased from Dako (Glostrup, Municipality, Denmark). HTR8/SVneo cells (CRL-3271, ATCC) were cultured as per instructions.

### 2.3. Immunohistochemistry and Immunocytochemistry

Human placental villous and decidua tissues were fixed in 10% formalin, embedded in paraffin, and sectioned at 4 *μ*m for immunohistochemistry staining. Sections were dewaxed, rehydrated, and subjected to antigen retrieval as previously optimized (10 mM sodium citrate for 5 min) [10]. After antigen retrieval, sections were treated with 3% hydrogen peroxide diluted in methanol to quench endogenous peroxidase. Sections were then rinsed with Tris-buffered saline (TBS) and blocked with 10% goat serum and 2% human serum in TBS (v/v) for 30 min at room temperature. Sections were then incubated with TPP1 antibody (0.14 *μ*g/mL) or isotype negative control (0.14 *μ*g/mL) at 4°C overnight [10]. To determine if TPP1 localizes to the EVTs, HLA-G was used as an EVT marker (1 *μ*g/mL) and serial sections at 2 *μ*m were used to localize TPP1 and HLA-G in the first trimester human decidua/implantation sites. Avidin/biotin reagents were used to reveal positive signaling, and sections were counterstained with hematoxylin to indicate cell nuclei. Staining intensity was scored by two scorers blinded to the patient characteristic, as previously described [12]. Briefly, a score of 0 denoted no positive staining and 3 was maximal staining. Intensity score took into account the number of stained cells and the intensity of staining in each cellular compartment. Groups were un-blinded after statistical analysis. For immunocytochemistry staining, 8-well chamber slides (#154534, Thermo) were used to culture HTR8/SVneo cells. Once confluent, cells were fixed in 4% paraformaldehyde and permeabilized with 0.1% Triton X-100 in TBS (v/v). Following TBS wash, cells were treated with 3% hydrogen peroxide and immunolabeled as described for placental sections.

### 2.4. In Vitro siRNA Transfection

Passaged HTR8/SVneo cells were counted and seeded into wells of 12-well plates with the goal to reach 70–80% confluency the next day. The cells were then washed with Dulbecco's phosphate-buffered saline (DPBS) and transfected with *TPP1* siRNA or scrambled negative control (20 nM) (Dharmacon, Lafayette, CO, USA) using the Lipofectamine RNAiMAX transfection system as instructed by the manufacturer. After 24 h, the transfection medium was replaced with fresh HTR8/SVneo cell culture medium and the cells were cultured for 48 h before being trypsinized, counted, and analyzed in real-time by xCELLigence assays and qPCR.

### 2.5. xCELLigence Assays

For HTR8/SVneo cell adhesion and proliferation, the transfected cells were trypsinized after 48 h of culture and seeded at 10,000 cells per well in the xCELLigence 96-well E-plate in fresh culture medium supplemented with 5% fetal bovine serum (FBS), as previously described [12]. The plate was monitored for cell index every 15 min for 8 h (for cell adhesion) and every 1 h following this for 72 h (for cell proliferation). HTR8/SVneo cell migration and invasion were measured using the CIM-plate 16 with 8 *μ*m pores (Roche, Sandhofer Straße, Mannheim, Germany). For migration, 40,000 transfected cells were resuspended in 5% FBS medium and seeded into the upper chambers. 10% FBS medium was added to lower chambers, as we previously described [12]. The cell index was recorded every 15 min for up to 18 h. Cell invasion was recorded using the same conditions as cell migration for up to 60 h with upper transwells being precoated with Matrigel (1:10 dilution in RPMI 1640). All data were calculated using the RTCA software 1.2 as supplied with the instrument and exported for statistical analysis.

### 2.6. RNA Isolation and RT-qPCR

The transfected HTR8/SVneo cells were subjected to RNA isolation using TRI Reagent after 48 h of culture. Genomic DNA contamination was removed using the TURBO DNA-free kit (#AM1907). After quantification using NanoDrop, 300 ng RNA was reversed transcribed into cDNA using the SuperScript™ III First-Strand Synthesis System (18080-051, Thermo). qPCR was performed on the Applied Biosystems ViiA7 system using the SYBR Green Master Mix (#4367659, Thermo) and specific primer pairs as summarized in Supplementary Table S1. Gene expression was normalized to *18S* and calculated by the comparative cycle threshold method (*ΔΔ*Ct).

### 2.7. Immunoblotting

The organic phase from TRI Reagent was collected for protein extraction as previously optimized [13]. Equivalent amounts of protein were boiled in lysis buffer (5% SDS, 20 mM EDTA, 140 mM NaCl, and 100 mM Tris) at 100°C for 5 min, prior to being resolved by SDS-PAGE (150 V, 90 min) and transferred to PVDF membranes (350 mA, 70 min). The membranes were blocked with 5% skim milk for 1 h at room temperature and incubated with TPP1 antibody (0.28 *μ*g/mL) at 4°C overnight. Following washing in TBS, the membranes were probed with HRP-conjugated secondary antibody and detected by chemiluminescence. GAPDH was reprobed, and appropriate protein band intensity was determined by densitometry using the ImageJ.

### 2.8. Statistics

Experiments were repeated at least four times with numbers indicated in each figure legend. Statistical analysis was performed using PRISM 8.0, two-tailed paired Student's *t*-test, or one-way or two-way ANOVA as appropriate with a significance threshold of *P* < 0.05. Data were presented as *mean* ± *SEM*.

## 3. Results

### 3.1. Expression of TPP1 in Human Placenta and Decidua

**Figure 1:**
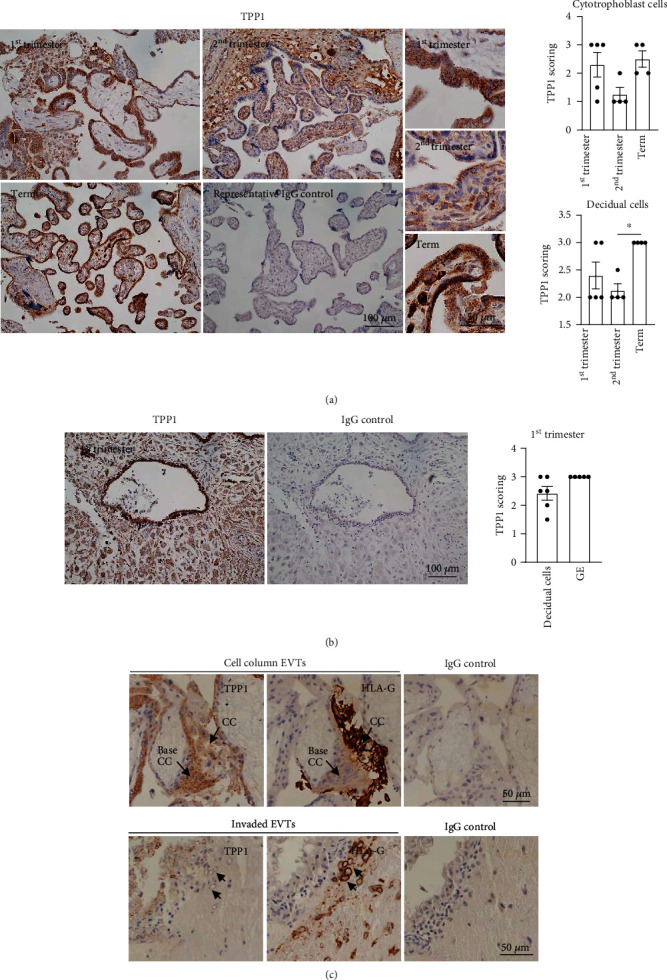
Immunolocalization of TPP1 in the human placenta throughout gestation and first trimester decidua. (a, b) Localization of TPP1 was examined in the human placenta (*n* = 4–5) and decidua (*n* = 6). TPP1 staining intensity in different cell types was scored blinded to the stage of pregnancy. (c) TPP1 co-localized with HLA-G in some EVTs with low expression in the human decidua (indicated by arrows, *n* = 4). All sections were counterstained with hematoxylin (blue) to indicate the cell nuclei. Immunostaining intensity scores were presented as mean ± SEM. ∗*P* < 0.05.CC: cell column, EVTs: extravillous trophoblast cells, GE: glandular epithelium.

We first sought to determine the relevance of TPP1 on placentation by assessing its expression and immunolocalization in human placenta throughout gestation and the first trimester decidua/implantation sites. In the placental villous, TPP1 exhibited discrete foci of cytoplasmic staining in syncytiotrophoblast, cytotrophoblast, and decidual cells across all trimesters of pregnancy (Figure 1(a) and Supplementary Figure S1). TPP1 immunostaining intensity scores in the decidual cells identified a significant increase (*P* < 0.05) between second-trimester and term placental villous (Figure 1(a)). TPP1 was also expressed in the decidual cells and glandular epithelial cells in the first trimester decidua/implantation sites (Figure 1(b)). To determine if TPP1 localizes to EVTs, we investigated TPP1 and HLA-G (EVT marker) staining on serial sections of the first trimester decidua basalis. TPP1 exhibited discrete foci of cytoplasmic staining at the base of the cell columns, and the staining intensity was apparently decreased in the HLA-G-positive EVTs that were further away from the cell columns (Figure 1(c)). In the EVTs that invaded into the maternal decidua, no positive staining of TPP1 was identified (Figure 1(c)). An IgG isotype control for TPP1 revealed no positive staining (Figure 1).

### 3.2. Knockdown of *TPP1* in HTR8/SVneo Cells Impaired Cell Adhesion and Proliferation

**Figure 2:**
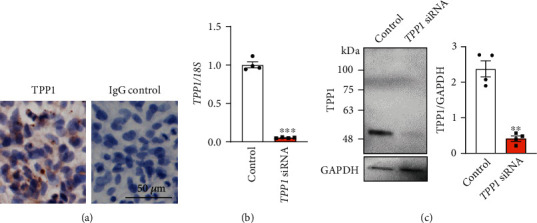
Examination of the efficiency of *TPP1* knockdown in HTR8/SVneo cells after siRNA treatment. (a) Localization of TPP1 in untreated HTR8/SVneo cells (*n* = 3). (b and c) Cells were transfected with *TPP1* siRNA or scrambled control, and after 48 h, qPCR (b) and immunoblotting (c) were used to determine *TPP1* expression. For qPCR, expression levels were normalized to *18S* (*n* = 4). *TPP1* was reduced by ~95% after siRNA treatment compared to control. For immunoblotting, blots were reprobed with GAPDH for densitometry analysis. (*n* = 4). ∗∗*P* < 0.01.

**Figure 3:**
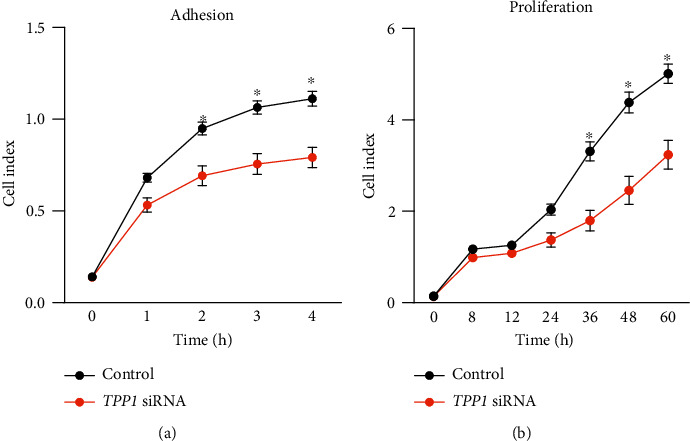
Real-time examination of the effects of *TPP1* knockdown on cell adhesion (a) and proliferation (b) of HTR8/SVneo cells via xCELLigence (expressed as cell index). (a) *TPP1* knockdown in HTR8/SVneo cells significantly decreased their adhesive capacity from 2 h to 4 h after seeding compared to control. (b) *TPP1* knockdown in HTR8/SVneo cells significantly decreased cell proliferation from 36 h to 60 h compared to control. Data were presented as mean ± SEM (*n* = 4). ∗*P* < 0.05.

**Figure 4:**
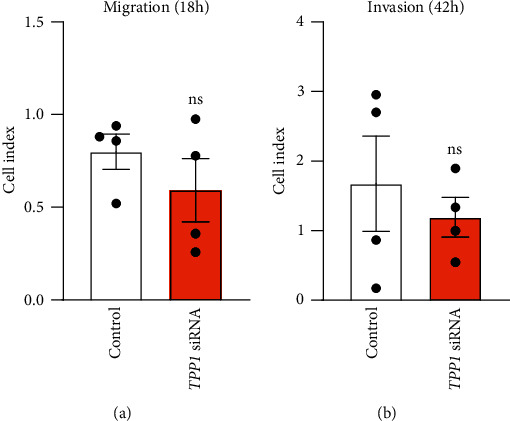
Examination of the effects of *TPP1* knockdown on cell migration (a) and invasion (b) of HTR8/SVneo cells via xCELLigence (expressed as cell index). No significant difference was identified for cell migration (at 18 h) and invasion (at 42 h) after *TPP1* knockdown compared to control. Data were presented as mean ± SEM (*n* = 4). ns: no significant difference.

To determine the function of TPP1 on placentation, HTR8/SVneo cells (first-trimester extravillous trophoblast-derived cell line) were transfected with *TPP1* siRNA to knock down the endogenous expression of TPP1 and cells were then monitored for adhesion, proliferation, migration, and invasion in real time via xCELLigence. Before use, we confirmed that HTR8/SVneo cells possess a conserved pattern of TPP1 expression compared to placental tissue (Figure 2(a)). qPCR confirmed that *TPP1* siRNA treatment in HTR8/SVneo cells resulted in ~95% decreased *TPP1* expression compared to control (*P* < 0.001; Figure 2(b)). The significant knockdown of TPP1 was also confirmed at the protein level by immunoblotting (*P* < 0.01; Figure 2(c)). Cell adhesion was significantly impaired after *TPP1* knockdown from 2 h to 4 h of culture, after which the cell index plateaued (*P* < 0.05; Figure 3(a)). Similarly, cell proliferation was significantly reduced from 36 h to 60 h of culture, after *TPP1* knockdown compared to control (*P* < 0.05; Figure 3(b)). HTR8/SVneo cell migration and invasion after *TPP1* knockdown were determined via xCELLigence assays, as previously described [12]. No significant changes in HTR8/SVneo cell migration and invasion between the *TPP1* siRNA treated group and control at 18 h and 42 h of culture were identified, respectively (Figure 4).

### 3.3. The Effect of *TPP1* Knockdown on the Expression of Genes Related to Cell Adhesion and Proliferation and TPP1 Functional Partners

**Figure 5:**
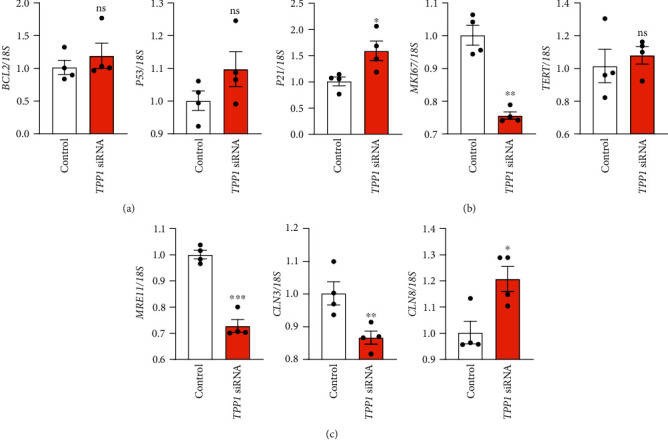
*TPP1* knockdown in HTR8/SVneo cells on the quantitative expression of target genes. The expression of genes related to cell adhesion (a), proliferation (b), and TPP1 target genes/functional partners (c) was examined by qPCR. Expression levels were normalized to *18S* (*n* = 4). Data were presented as mean ± SEM. ∗*P* < 0.05, ∗∗*P* < 0.01, ∗∗∗*P* < 0.001, ns: no significant difference.

The expression of two adhesion-related genes B-cell lymphoma 2 (*BCL2*) and *P53*, as we previously identified in Ishikawa cells after *TPP1* knockdown [10], was determined by qPCR. No significant changes were identified between groups (Figure 5(a)). With relevance to cell proliferation, among three genes determined, *TPP1* knockdown significantly increased *P21* expression (*P* < 0.05) and decreased marker of proliferation Ki-67 (*MKI67*) expression (*P* < 0.01), compared to control, respectively (Figure 5(b)). No significant difference was identified for the expression of telomerase reverse transcriptase (*TERT*) between groups (Figure 5(b)). The expression of TPP1 functional partners/targets was also examined in the HTR8/SVneo cells following *TPP1* knockdown by qPCR. *TPP1* knockdown significantly decreased the expression of *MRE11* (*P* < 0.001) and ceroid-lipofuscinosis, neuronal (*CLN*) 3 (*P* < 0.01) while the expression of *CLN8* was significantly increased (*P* < 0.05) compared to control (Figure 5(c)).

## 4. Discussion

This study determined the localization of TPP1 in the human placenta throughout gestation and the first-trimester decidua/implantation sites. TPP1 showed discrete foci of cytoplasmic staining in the syncytiotrophoblast, cytotrophoblast, and decidual cells; however, minimal/no staining was identified in the HLA-G-positive EVTs, especially in the EVTs that invaded into the decidua. Functional analysis revealed a role for TPP1 in regulating trophoblast cell adhesion and proliferation, but not migration and invasion.

After blastocyst implantation, the cytotrophoblast cells proliferate to form cell columns based at the anchoring villi which then further differentiate to a less proliferative but more invasive phenotype in order to invade into the maternal decidua [1, 3]. Dysregulation of this process may lead to obstetric complications such as preeclampsia due to inadequate placentation [1, 14]. Our immunohistochemistry staining data confirmed the expression of TPP1 in these proliferative cells in the cell columns, suggesting a direct role of TPP1 in regulating trophoblast cell proliferation. In support of this hypothesis, knockdown of *TPP1* in the HTR8/SVneo cells significantly impaired cell proliferation. We also identified an inverse relationship between TPP1 and HLA-G staining in the first trimester human decidua. While the low levels of TPP1 were still identified in the HLA-G-positive EVTs residing at the placental cell columns, no TPP1 staining was revealed in the invaded HLA-G positive EVTs in the decidua where cells are no longer proliferative [2]. Consistently, knockdown of *TPP1* in the HTR8/SVneo cells did not compromise cell invasion, suggesting that TPP1 may not be involved in regulating trophoblast cell invasion.

MRE11 has been recently identified as a TPP1 downstream target [15]. In addition to enhancing DNA repair and improving cell survival [15], MRE11 is also required for cell proliferation [16]. In human breast cancer cells, MRE11 regulates cell proliferation via signal transducer and activator of transcription 3 (STAT3) [17]. As a downstream target of TPP1, *MRE11* expression was significantly decreased after *TPP1* knockdown in the HTR8/SVneo cells. Although we remain uncertain if MRE11 is involved in regulating HTR8/SVneo cell proliferation, the effects of *TPP1* knockdown on HTR8/SVneo cell proliferation were confirmed by two other proliferation markers *MKI67* and *P21*. P21 is a cell-cycle inhibitory protein that leads to cell-cycle arrest in the S phase by targeting cyclin-CDK complexes [18]. The expressional changes of P21 in relation to cell proliferation have been confirmed in the HTR8/SVneo cells, and in this study, we identified similarly that cells with defects in proliferation had higher *P21* expression levels, compared to control [18].

We have previously shown that in human endometrial epithelial cells, knockdown of *TPP1* impairs cell adhesion by targeting BCL2 and P53 [10]. Although *TPP1* knockdown similarly affected cell adhesion in the HTR8/SVneo cells, no significant changes were recorded on the expression of BCL2 and P53, suggesting cell type-specific regulation of targeted genes to control cell adhesion. We have also reported that TPP1 knockdown in primary human endometrial stromal cells does not impact decidualization or expression of decidualization markers [10]. In this study, TPP1 was detected at appreciable levels in the decidual cells across all trimesters of pregnancy, with a lower level which has been observed in the second trimester. However, the function of TPP1 in decidual cells was not determined. It is known that decidual cells can regulate trophoblastic invasion by secreting factors locally [19]. Although TPP1 was not detected in invaded EVTs in the decidua and knockdown of TPP1 in HTR8/SVneo cells did not impact cell invasion, we cannot exclude a potential indirect regulation of EVT invasion via decidual cells.

## 5. Conclusions

Our study identified that *TPP1* knockdown in HTR8/SVneo cells impaired cell proliferation, as evidenced by both xCELLigence analysis and effects on the expression of proliferation markers *MKI67* and *P21*. It remains to be determined if TPP1 dysregulation in the first-trimester placenta of women causes obstetric complications.

## Data Availability

All data are provided in the submitted manuscript.

## Abbreviations

EVT: Extravillous trophoblast
TPP1: Tripeptidyl peptidase 1
CLN2: Neuronal ceroid lipofuscinosis type 2
TBS: Tris-buffered saline
DPBS: Dulbecco's phosphate-buffered saline
FBS: Fetal bovine serum
BCL2: B-cell lymphoma 2
MKI67: Marker of proliferation Ki-67
TERT: Telomerase reverse transcriptase
CLN: Ceroid-lipofuscinosis, neuronal
STAT3: Signal transducer and activator of transcription 3.

## Supplementary Materials

Supplementary 1 Supplementary Figure S1. Immunolocalization of TPP1 in the decidual cells throughout gestation. The immunolocalization of HLA-G was also determined in the first trimester decidua using serial sections to distinguish EVTs.

Supplementary 2 Supplemental Table 1. Primers used throughout this study.
