# Supplementary figures and images for: Tripeptidyl Peptidase 1 Regulates Human Trophoblast Cell Proliferation Implying a Role in Placentation
Source: Biomed Res Int. 2022 Sep 12;2022:6856768. doi: 10.1155/2022/6856768 (PMC9485709; doi:10.1155/2022/6856768)

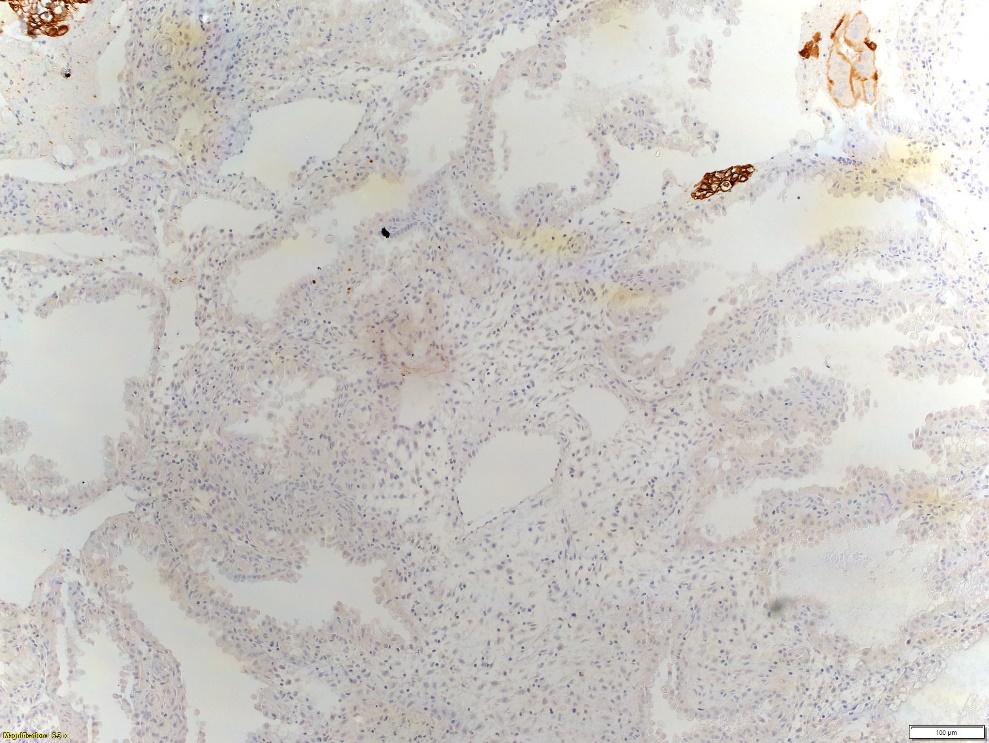

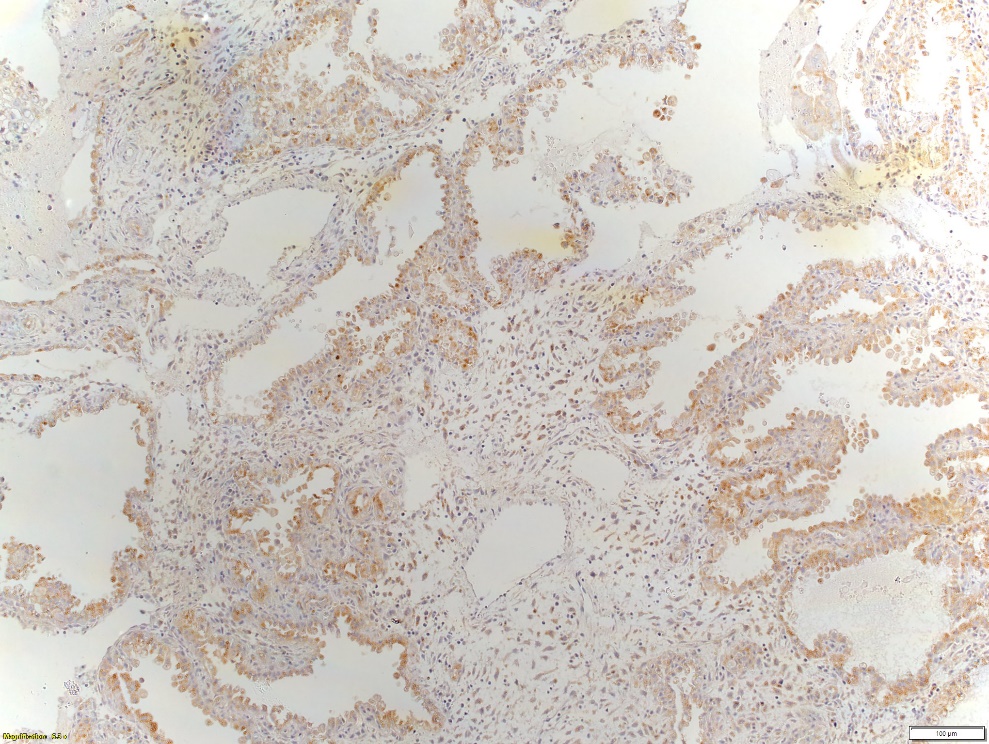


**1st trimester**

**TPP1**

**HLA-G**

**2nd trimester**

**Term**

**Supplementary Figure 1**


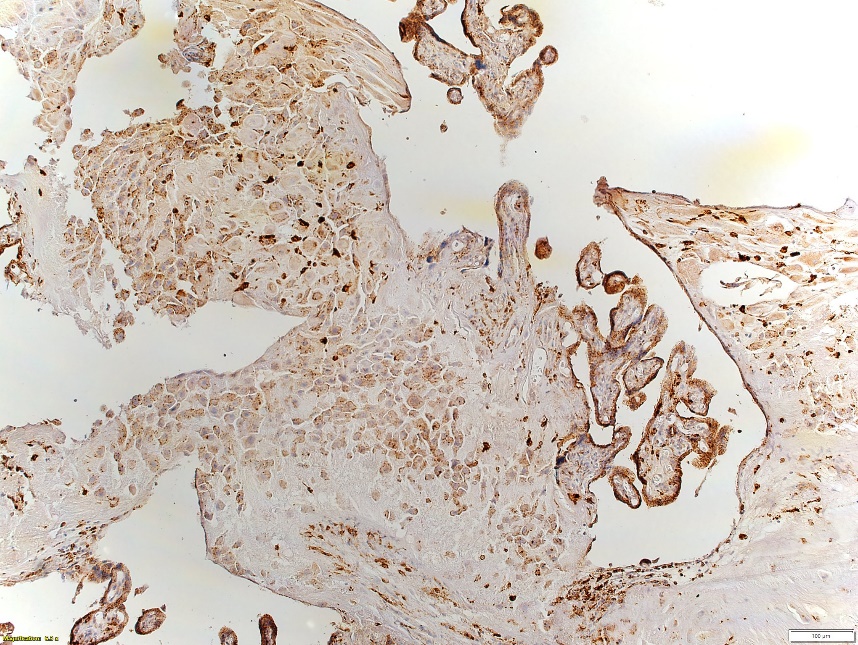


**100 µm**


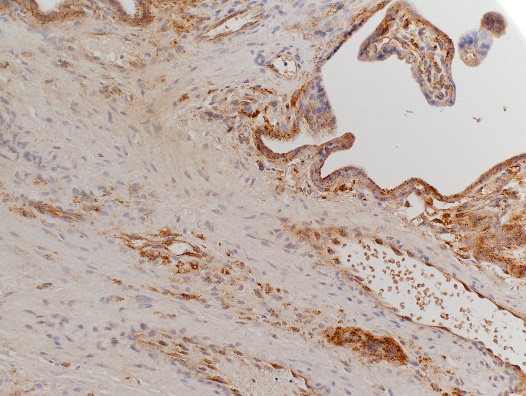


**TPP1**

**TPP1**

Supplement: Supplementary 1 — Supplementary Figure S1. Immunolocalization of TPP1 in the decidual cells throughout gestation. The immunolocalization of HLA-G was also determined in the first trimester decidua using serial sections to distinguish EVTs. [file 6856768.f1.docx]
