# Supplementary material for: Tripeptidyl Peptidase 1 Regulates Human Trophoblast Cell Proliferation Implying a Role in Placentation
Source: Biomed Res Int. 2022 Sep 12;2022:6856768. doi: 10.1155/2022/6856768 (PMC9485709; doi:10.1155/2022/6856768)
Supplement: Supplementary 2 — Supplemental Table 1. Primers used throughout this study. [file 6856768.f2.docx]

| **Supplemental Table 1. Primers used throughout this study** | | |
| --- | --- | --- |
| **Gene** | **Forward (5'-3')** | **Reverse (5'-3')** |
| *18S* | GATCCATTGGAGGGCAAGTCT | CCAAGATCCACCTACGAGCTT |
| *TPP1* | CCTCCACACGGTGCAAAAATG | CTCTGCTTGTCGGATGCTCAG |
| *P21* | TGTCCGTCAGAACCCATGC | AAAGTCGAAGTTCCATCGCTC |
| *CLN8* | TGGTCGCTGGCTTTGTCTTC | AGAACGGTAAGTGGCATTCAG |
| *MKI67* | AGAAGAAGTGGTGCTTCGGAA | AGTTTGCGTGGCCTGTACTAA |
| *P53* | CCCAAGCAATGGATGATTTGA | GGCATTCTGGGAGCTTCATCT |
| *TERT* | AAATGCGGCCCCTGTTTCT | CAGTGCGTCTTGAGGAGCA |
| *BCL2* | GATTGTGGCCTTCTTTGAG | GTTCCACAAAGGCATCC |
| *CLN3* | CGCCCACGACATCCTTAGC | AGCAGCCGTAGAGACAGAGTT |
| *MRE11* | ATGCAGTCAGAGGAAATGATACG | CAGGCCGATCACCCATACAAT |
